# Evaluation of alternative prognostic thresholds for SP142 and 22C3 immunohistochemical PD-L1 expression in triple-negative breast cancer: results from a population-based cohort

**DOI:** 10.1007/s10549-024-07561-x

**Published:** 2024-12-10

**Authors:** Gudbjörg Sigurjonsdottir, Tommaso De Marchi, Anna Ehinger, Johan Hartman, Susann Ullén, Karin Leandersson, Ana Bosch, Johan Staaf, Fredrika Killander, Emma Niméus

**Affiliations:** 1 Division of Oncology, Department of Clinical Sciences Lund, Lund University, Sölvegatan 19 - BMC F12, 221 84 Lund, Sweden; 2 Department of Hematology, Oncology and Radiation Physics, Skåne University Hospital, Skåne, Sweden; 3 Department of Clinical Genetics, Pathology and Molecular Diagnostics, Laboratory Medicine, Region Skåne, Lund, Sweden; 4 Department of Oncology and Pathology, Karolinska Institute and University Hospital, Stockholm, Sweden; 5 Clinical Studies Sweden, Skåne University Hospital, Lund, Sweden; 6 Cancer Immunology, Department of Translational Medicine, Clinical Research Center, Lund University, Malmö, Sweden; 7 Division of Translational Cancer Research, Department of Laboratory Medicine, Lund University, Medicon Village, Lund, Sweden; 8 Divison of Surgery, Department of Clinical Sciences Lund, Lund University, Lund, Sweden; 9 Department of Surgery, Skåne University Hospital, Malmö, Sweden

**Keywords:** Triple-negative breast cancer, PD-L1, SP142, 22C3, Tumor infiltrating lymphocytes, Gene expression

## Abstract

**Background:**

Immune checkpoint inhibitors are now a part of the treatment arsenal for triple-negative breast cancer (TNBC) but refinement of PD-L1 as a prognostic and predictive biomarker is a clinical priority. We aimed to evaluate the relevance of novel PD-L1 immunohistochemical (IHC) thresholds in TNBC with regard to PD-L1 gene expression, prognostic value, tumor infiltrating lymphocytes (TILs), and TNBC molecular subtypes.

**Material & methods:**

PD-L1 was scored in a tissue microarray with the SP142 (immune cell (IC) score) and the 22C3 (combined positive score; CPS) IHC assays and TIL abundance evaluated in whole slides in a population-based cohort of 237 early-stage TNBC patients. Survival analysis was performed and RNA sequencing data employed for molecular profiling.

**Results:**

As expected, PD-L1 positivity (IC ≥ 1% and/or CPS ≥ 1) was significantly associated with better prognosis compared to zero PD-L1 expression. Importantly however, also patients with intermediate expression (IC > 0%, < 1%; CPS > 0, < 1) showed a trend toward improved outcome. Tumors with intermediate PD-L1 IHC expression also had intermediate PD-L1 (*CD274*) gene expression (mRNA). Patients who were both low in TILs (< 30%) and PD-L1 (IC < 1%; CPS < 1) tended to have the poorest prognosis.

PD-L1 positive tumors clustered significantly more often as Immunomodulatory-high and Basal-Like 1-high TNBC molecular subtypes and were enriched in immune response and cell cycle/proliferation signaling pathways. PD-L1-zero tumors on the other hand were enriched in cell growth, differentiation, and metastatic potential pathways and clustered more prevalently as Luminal-Androgen-Receptor-high and Mesenchymal-high. PD-L1-intermediate tumors categorized with neither PD-L1-positive nor PD-L1-zero tumors on the hierarchical clustering level, consigning them as a unique subgroup.

**Conclusion:**

With both SP142 and 22C3, we identified an intermediate IHC PD-L1 group within TNBCs that was supported on the molecular level. Any PD-L1 IHC expression, even though it is < 1, tended to have positive prognostic impact. We suggest that the generally accepted threshold of PD-L1 IHC positivity in TNBC should be investigated further.

The Swedish Cancerome Analysis Network – Breast (SCAN-B) study was retrospectively registered 2nd Dec 2014 at ClinicalTrials.gov; ID NCT02306096.

**Supplementary Information:**

The online version contains supplementary material available at 10.1007/s10549-024-07561-x.

## Introduction

Improved treatments for triple-negative breast cancer (TNBC) are urgently required in the attempt to achieve more favorable prognosis. Immune checkpoint inhibition targeting the PD-1/PD-L1 (Programmed Death-Ligand 1) axis has now become a part of the therapeutic arsenal for early-stage and metastatic TNBC. However, deeper understanding of PD-L1 as a prognostic and predictive biomarker is needed and refinement of predictive biomarkers for this therapy option is crucial [1, 2].

In the metastatic TNBC setting, the PD-L1 inhibitor atezolizumab and the PD-1 inhibitor pembrolizumab have shown meaningful survival benefit in combination with chemotherapy (CT) in patients with SP142 PD-L1 expressing immune cells (ICs) of ≥ 1% and 22C3 combined positive score (CPS) of ≥ 10, respectively [3–6]. In early-stage TNBC phase III trials, improved patient outcome with the addition of atezolizumab or pembrolizumab to CT has been found irrespective of PD-L1 status [7–9]. Immunohistochemical (IHC) PD-L1 expression is currently the only clinically approved predictive biomarker for checkpoint inhibition in TNBC and it has been suggested to implement combined evaluation of PD-L1 and stromal tumor infiltrating lymphocytes (TILs) as a more comprehensive biomarker for patient selection [10]. PD-L1 positivity and TIL abundance are positively correlated [11] but their prognostic and predictive interaction might be more complex. For example, in the IMpassion130 trial, improved outcomes with atezolizumab in TIL positive tumors were observed only if they were also PD-L1 positive, concluding that PD-L1 expression in ICs was a more robust predictive biomarker for checkpoint inhibition than TILs alone [12]. Hence, PD-L1 and TILs seem to be a limited surrogate marker of the immune activity of the microenvironment. Moreover, TNBC is a highly heterogeneous disease consisting of several subtypes, each with its own molecular characteristics. Molecular profiling, by e.g., RNA sequencing (RNAseq), may possibly assist in identifying genetic alterations or active molecular pathways that could reveal mechanisms involved in these complex immunological interactions [2, 13].

It is of clinical importance not missing out potential checkpoint blockade responders, as well as avoiding treating patients that are not likely to benefit. TNBC tumors with zero IHC PD-L1 expression and over zero but not reaching the threshold of one have to our best knowledge clinically been pooled together as PD-L1-negative (IC < 1%; CPS < 1). Therefore, in the current study, we evaluated the impact of alternatively defined SP142 and 22C3 PD-L1 expression levels (zero, intermediate (SP142 > 0% but < 1%; 22C3 CPS > 0, < 1) and positive (IC ≥ 1%; CPS ≥ 1)) in early-stage TNBC patients, investigated the prognostic role of PD-L1 in relation to TILs, and aimed to identify molecular profiles in the context of these PD-L1 expression categories by employing RNAseq data.

## Material and methods

### Study population

**Fig. 1:**
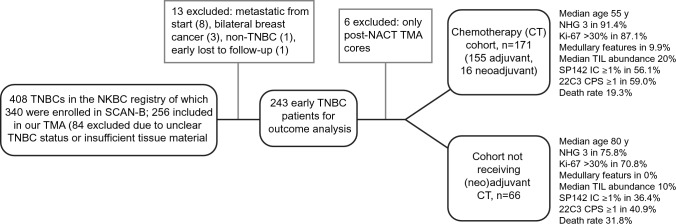
Study flowchart. Our final cohort consisted of 237 early-stage TNBC patients from the population-based SCAN-B cohort. Clinicopathological characteristics that significantly differed between the patients receiving (neo)adjuvant chemotherapy and those not receiving are shown to the right and in more detail in Table S1. Abbreviations: *TNBC* Triple-negative breast cancer; *NKBC* National Breast Cancer Quality (NKBC) registry; *SCAN-B* Swedish Cancerome Analysis Network – Breast; *TMA* Tissue microarray; *NACT* Neoadjuvant chemotherapy; *CT* Chemotherapy; *NHG* Nottingham histologic grade; *TILs* Tumor infiltrating lymphocytes; IC PD-L1 expression on immune cells; *CPS* Combined positive score

The cohort has been described in detail previously [14] and consists of patients diagnosed with primary TNBC in Region Skåne, Sweden, between 2010 and 2015 who were identified (*n* = 408) in the Swedish National Breast Cancer Quality (NKBC) registry and enrolled (*n* = 340) in the Swedish Cancerome Analysis Network – Breast (SCAN-B) study (ClinicalTrials.gov ID NCT02306096) [15]. Exclusion criteria (*n* = 103) were unclear TNBC status, insufficient tissue material, metastatic disease at diagnosis or prior to start of chemotherapy (CT), bilateral breast cancer, loss to follow-up before treatment start or tissue microarray (TMA) cores only available from residual disease after neoadjuvant CT. 237 patients remained in the final cohort of which 171 received CT (155 adjuvant and 16 neoadjuvant; the CT-cohort) and 66 received no (neo)adjuvant CT (the non-CT-cohort). The patients were not treated with checkpoint inhibitors. Study flow chart is shown in Fig. 1 along with clinicopathological features that significantly differed between the CT- and non-CT-cohort (clinicopathological features also available in Table S1). RNA sequencing data for gene expression profiling (GEX) were available for 82% of the patients (194/237) from the SCAN-B consortium [15] and has previously been published [14].

### Evaluation of PD-L1 and TILs

PD-L1 was assessed immunohistochemically with the SP142 anti-PD-L1 antibody clone on a Ventana BenchMark Ultra platform (Ventana Medical Systems, Inc., AZ, U.S) and with the 22C3 clone on a Dako Autostainer Link 48 platform (Agilent, Inc., CA, U.S) in formalin-fixed, paraffin-embedded tumor samples in a TMA where each sample was presented in two 1.0 mm diameter TMA cores. Preparations and stainings were done according to the manufacturer´s instructions. PathXL Philips Xplore (Koninklijke Philips N.V., NL) was used to visually assess the TMA images. With the SP142 assay, PD-L1 was evaluated as the percentage of tumor area occupied by PD-L1 stained immune cells (% ICs). With the 22C3 assay, PD-L1 expression was evaluated as combined positive score (CPS) which is the combined number of PD-L1 stained tumor cells (TCs), TILs, and macrophages divided by the total number of TCs, multiplied with 100. The TMA core with highest score was set as the respective CPS and IC + score for the tumor. Thresholds for SP142 IC (of note, as %) and 22C3 CPS were evaluated at ≥ 1 (positive), over zero but less than one (intermediate, > 0 but < 1), and at zero (0).

Stromal TIL abundance was evaluated according to the international TIL working group in a light microscope on a hematoxylin–eosin stained whole slide sections, and not in a TMA as opposed to our PD-L1 scoring, as percentage of TILs covering the tumoral stromal area [16] (https://www.tilsinbreastcancer.org/). The average score was used if a patient had more than one evaluable slide and we chose a threshold of 30% for high versus low TILs as performed in a previous early-stage TNBC study [17]. For both PD-L1 and TILs, scoring was performed on samples from the surgical specimen before eventual CT (patients treated with adjuvant CT and patients not treated with CT) and on the pre-CT core needle biopsy for the patients receiving neoadjuvant CT.

### Statistical methods

Statistical analyses were performed with the SPSS 26.0 software package. All analyses in the study were performed separately for SP142 and 22C3 PD-L1 status. Survival analyses were performed with the Kaplan–Meier method and with Cox regression along with hazard ratios (HR) and 95% confidence intervals (CIs). HR was interpreted as average over time if the proportional hazard assumption was not fulfilled. Overall survival (OS), invasive disease-free survival (IDFS), and distant relapse-free interval (DRFI) were defined as endpoints in survival analyses with support of the STEEP criteria [18] as described in our previous study [19]. Chi-square test was applied to compare categorical values between groups and nonparametric Mann–Whitney or Kruskal Wallis test when comparing non-categorical values between two or three groups, respectively. The strength of association between categorical and continuous variables was estimated with the Spearman method and visualized with boxplots. A p value less than 0.05 was considered statistically significant and all p-tests were two sided.

### Analyses of RNA sequencing data

Analyses of RNAseq data for GEX profiling were performed with the R software (v 3.6.1). RNAseq and patient data were matched resulting in a list of 16,258 genes of 194 samples. FKPM (Fragment Per Kilobase Million) values were Log2-transformed, imputed (missing data to 0), mean-centered, and scaled (samples and genes). Transcript abundance derived from RNAseq data was employed to classify tumors into TNBC subtypes/clusters according to Lehmann et al. [13] where TNBC subtypes were assigned to each sample using the STROMA4 (v 1.14.0) package in R (v 4.0.5). Differential expression of genes between different IHC PD-L1 groups was performed by running Limma on FKPM values and the Benjamini–Hochberg method was employed to adjust the resulting p values for multiple testing. Gene Set Enrichment Analysis (GSEA) was employed to derive enriched pathways in PD-L1-defined sample groups with normalized enrichment score [20]. Data were queried against the Hallmarks database (v 5.2), permutation type was set to gene set, weighted scoring was enabled, and t test was selected as metric. Default settings were kept for all other parameters. False discovery rate (FDR) < 0.25 was set as cut-off for pathway significance.

## Results

### Association of PD-L1 IHC expression with clinicopathological features, TILs, and PD-L1 mRNA levels

In the overall cohort (*n* = 237), 34.2% of tumors (81 out of 237) scored IC 0% with the SP142 assay, 15.2% (36/237) were > 0% but < 1% (SP142-intermediate), and 50.6% (120/237) were IC ≥ 1% (SP142-positive). With the 22C3 assay, 23.3% (54/232) had CPS 0 (22C3-zero), 22.8% (53/232) were > 0 but < 1 (22C3-intermediate) and 53.9% (125/232) had CPS ≥ 1 (22C3-positive, of which 63 had CPS ≥ 10). Five tumors were missing for the 22C3 staining due to unevaluable TMA cores (Table 1).

**Table 1:** PD-L1 immunohistochemical staining results with SP142 (rows) and 22C3 (columns) in the overall cohort

	**22C3 CPS 0**	**22C3 CPS > 0, < 1**	**22C3 CPS ≥ 1**	*total*
**SP142 IC 0%**	51	22	6	79
**SP142 IC > 0%, < 1%**	1	15	19	35
**SP142 IC ≥ 1%**	2	16	100	118
*total*	54	53	125	232

Five tumors were missing for 22C3 due to unevaluable TMA cores. Two of these were SP142 zero respective positive (≥ 1%) and one was SP142-intermediate (> 0%, < 1%). Sixty-three of the 125 CPS ≥ 1 tumors were CPS ≥ 10

**Fig. 2:**
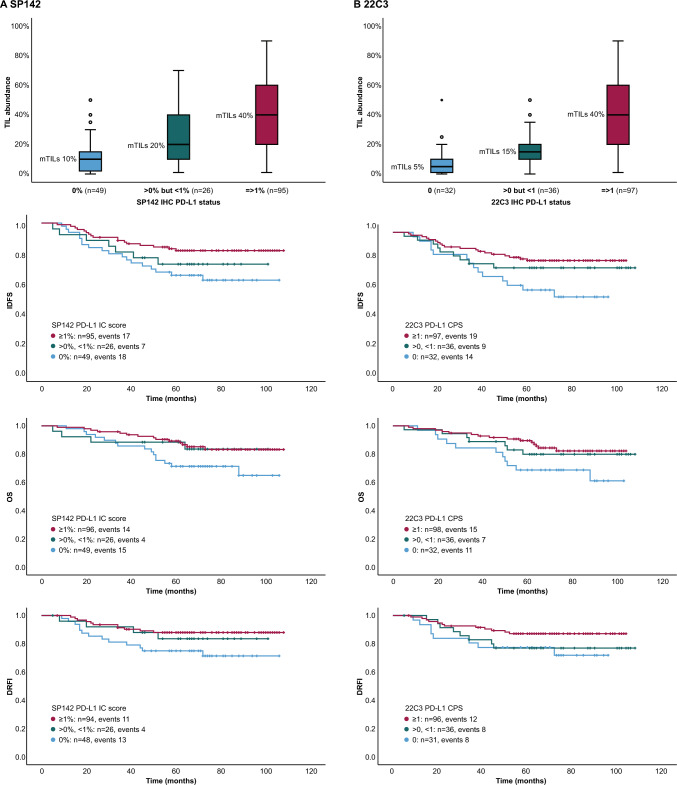
Abundance of tumor infiltrating lymphocytes (TILs) and Kaplan–Meier survival analyses according to PD-L1 status in the cohort receiving (neo)adjuvant chemotherapy. In panel (**A**) boxplots for the association of TIL abundance and PD-L1, invasive disease-free survival (IDFS), overall survival (OS), and distant relapse-free interval (DRFI) for SP142 PD-L1 expression (log rank p value 0.050, 0.076 and 0.069, respectively) and in panel (**B**) for 22C3 (log rank p value 0.030 for IDFS, 0.073 for OS and 0.151 DRFI). Corresponding Cox regression analyses are shown in Table 2. Abbreviations: *mTILs* Median TIL abundance; *IHC* Immunohistochemistry; *IC* Immune cell; *CPS* Combined positive score

We next analyzed clinicopathological characteristics according to the three PD-L1 categories (zero, intermediate, positive) in the overall cohort. In our previous study, we found that IHC PD-L1 expression at SP142 IC 1% and 22C3 CPS 1 cut-offs was positively associated with TIL abundance, Nottingham histologic grade, Ki-67 proliferation index, and medullary histological features [19]. In the present study, we found that the intermediate PD-L1 group did not consequently categorize with either the PD-L1-zero or the PD-L1-positive groups regarding histologic grade, Ki-67 or TIL abundance (Table S2). In the CT-cohort, TIL abundance was 40%, 20%, and 10% for the SP142-positive, SP142-intermediate, and the SP142-zero group, respectively. For 22C3, the abundance of TILs was 40%, 15%, and 5% for the positive, intermediate, and the zero group, respectively (boxplots in Fig. 2).

**Fig. 3:**
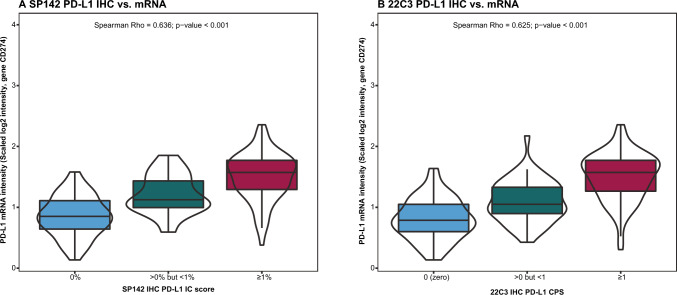
Association of PD-L1 immunohistochemical (IHC) categories (zero, intermediate (> 0, < 1), positive (≥ 1); x-axis) and PD-L1 (*CD274*) gene expression (mRNA; y-axis) in the overall cohort. In (**A**) for the SP142 IHC assay and in (**B**) for 22C3. Abbreviations: *IC* Immune cell; *CPS* Combined positive score

The association between IHC PD-L1 categories and PD-L1 (*CD274*) gene expression (mRNA) levels revealed a significant positive stepwise association, with Spearman correlation coefficient of 0.64 and 0.63 for SP142 and 22C3, respectively (p values < 0.001). Consistent with the IHC group definition, the IHC zero groups showed the lowest *CD274* mRNA expression, followed by the intermediate groups and IHC positive groups (Fig. 3).

### Impact of the alternative PD-L1 categories on patient outcome in the CT-cohort

We continued to perform outcome analyses according to the three IHC expression groups in the (neo)adjuvant-CT-cohort (Table 2 and survival charts in Fig. 2). The reason to this is that we previously [19] found that PD-L1 and TILs as binary variables had significant prognostic impact only in the (neo)adjuvant-CT-cohort and not in the non-CT-cohort. For both SP142 and 22C3, the PD-L1-zero groups tended to have the worst prognosis. PD-L1 positivity and intermediate expression were significantly and non-significantly, respectively, associated with improved outcome for all the clinical endpoints when compared to the PD-L1-zero groups (HRs ranged from 0.40 to 0.45 in favor of the positive groups; all p values < 0.05, and HRs ranged from 0.51 to 0.84 in favor of the intermediate groups, depending on IHC antibody and outcome, non-significant). For SP142 OS and DRFI, and 22C3 IDFS and OS, the PD-L1 intermediate groups tended to prognostically resemble the positive subgroups more than the zero categories, as reflected in smaller differences in HRs between the positive and intermediate versus the intermediate and zero PD-L1 groups (Table 2; the regression analyses where the intermediate group is set as references).

**Table 2:** Cox regression analyses according to PD-L1 status in the cohort receiving (neo)adjuvant chemotherapy

	**IDFS** (events = 42)		**OS** (events = 33)		**DRFI** (events = 28)	
**SP142 status**	HR (95% CI)	p- value	HR (95% CI)	p- value	HR (95% CI)	p- value
≥ 1% (positive)	0.45 (0.23–0.87)	0.017	0.45 (0.22–0.93)	0.032	0.40 (0.18–0.90)	0.027
> 0% < 1% (intermediate)	0.73 (0.30–1.74)	0.475	0.51 (0.17–1.55)	0.238	0.56 (0.18–1.71)	0.306
*0% (zero)*	*Ref*		*Ref*		*Ref*	
-						
≥ 1% (positive)	0.61 (0.25–1.48)	0.277	0.87 (0.29–2.66)	0.813	0.72 (0.23–2.27)	0.580
> *0%* < *1% (intermediate)*	*Ref*		*Ref*		*Ref*	
0% (zero)	1.38 (0.57–3.29)	0.475	1.94 (0.65–5.86)	0.238	1.80 (0.59–5.51)	0.306
-						
≥ *1% (positive)*	*Ref*		*Ref*		*Ref*	
> 0% < 1% (intermediate)	1.63 (0.68–3.93)	0.277	1.14 (0.38–3.48)	0.813	1.38 (0.44–4.34)	0.580
0% (zero)	2.24 (1.15–4.35)	0.017	2.22 (1.07–4.61)	0.032	2.48 (1.11–5.54)	0.027
-						
**22C3 status**	-					
≥ 1 (positive)	0.41 (0.20–0.81)	0.010	0.42 (0.19–0.91)	0.028	0.45 (0.18–1.10)	0.080
> 0, < 1 (intermediate)	0.55 (0.24–1.28)	0.164	0.54 (0.21–1.39)	0.199	0.84 (0.32–2.24)	0.730
*0 (zero)*	*Ref*		*Ref*		*Ref*	
-						
≥ 1 (positive)	0.73 (0.33–1.62)	0.445	0.78 (0.32–1.90)	0.578	0.53 (0.22–1.31)	0.170
> *0,* < *1 (intermediate)*	*Ref*		*Ref*		*Ref*	
0 (zero)	1.81 (0.78–4.19)	0.164	1.86 (0.72–4.80)	0.199	1.12 (0.45–3.17)	0.730
-						
≥ *1 (positive)*	*Ref*		*Ref*		*Ref*	
> 0, < 1 (intermediate)	1.36 (0.62–3.01)	0.445	1.29 (0.53–3.17)	0.578	1.87 (0.77–4.58)	0.170
0 (zero)	2.47 (1.24–4.92)	0.010	2.40 (1.10–5.23)	0.028	2.23 (0.91–5.45)	0.080

*Ref.:* Either the PD-L1-zero, intermediate or positive groups are set as references. Corresponding Kaplan–Meier curves are shown in Fig. 3

### Effect of PD-L1 and TILs in combination on patient outcome in the CT-cohort

**Fig. 4:**
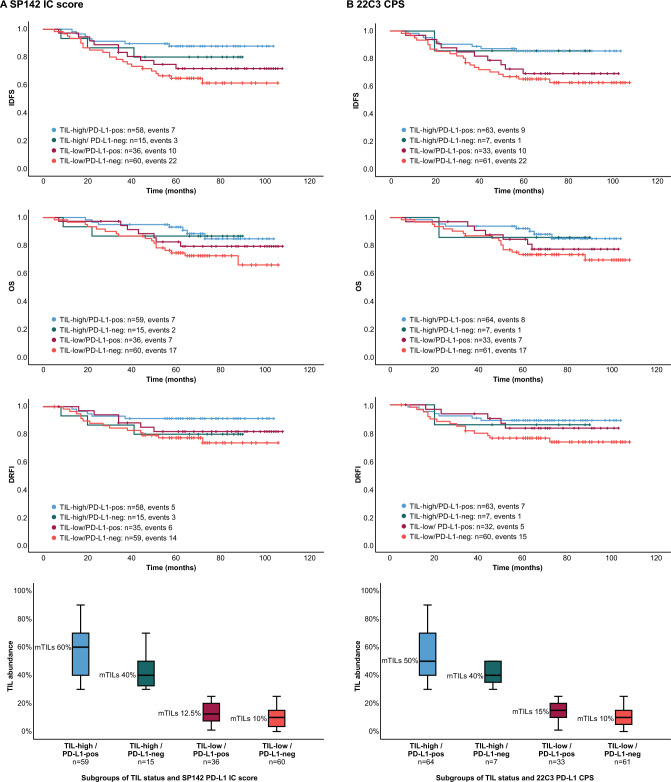
Kaplan–Meier estimates and abundance of tumor infiltrating lymphocytes (TILs; boxplots) in the cohort receiving (neo)adjuvant chemotherapy according to four combined subgroups of PD-L1 and TIL status. Invasive disease-free survival (IDFS), overall survival (OS), and distant relapse-free interval (DRFI) to differentiate the prognostic impact of PD-L1 status separately in cases that are high in TILs (≥ 30%; blue and green curves) and low in TILs (< 30%; red and orange). In panel (**A**) for SP142 IC < 1% (neg.) versus ≥ 1% (pos.; log rank p value 0.024 for IDFS, for OS 0.139 and 0.179 for DRFI) and in panel (**B**) for 22C3 CPS < 1 vs. ≥ 1 (log rank p value 0.045 for IDFS, for OS 0.216 and 0.225 for DRFI). Corresponding Cox regression analyses are shown in Table 3. Abbreviations: *mTILs* Median TIL abundance; *IC* Immune cell; *CPS* Combined positive score

We also investigated the prognostic impact of PD-L1 status separately in cases that are high in TILs and low in TILs (< 30% vs ≥ 30%). This was performed according to binary PD-L1 expression status (SP142 IC at a threshold of 1% and 22C3 CPS at a cut-off of 1; Table 3 and Fig. 4). In the TIL-low groups, PD-L1 positivity had a non-significant tendency for positive effect on outcome compared to PD-L1 negativity (HRs ranging from 0.59 to 0.79 depending on IHC assay and clinical endpoint). Patients with tumors that were low in TILs and also PD-L1 negative tended to have the poorest prognosis overall, which was significant when compared to the TIL-high/PD-L1-pos groups (HRs ranging from 2.37 to 3.45, depending on IHC assay and clinical endpoint, all p values < 0.05) and non-significant when compared to the TIL-high/PD-L1-neg or the TIL-low/PD-L1-pos groups (Table 3). In the TIL-high cohort, PD-L1 positivity also had a non-significant tendency for positive effect on outcome compared to PD-L1 negativity (HRs ranging from 1.03 to 2.46 depending on IHC antibody assay and clinical endpoint, in favor of PD-L1 positivity). However, the PD-L1 positive cases had higher median TIL abundance than the PD-L1 negative cases and it was most pronounced in the TIL-high cohort (Fig. 4; boxplots). Therefore, since TILs are known to be a positive prognostic factor in TNBC, we cannot directly assess the impact of PD-L1 per se in the TIL-high cohort. The subgroups became too small if employing this analysis with the three categorical PD-L1 status (zero, intermediate, positive) in combination with binary TIL status, and was therefore not performed.

**Table 3:** Cox regression analyses in the chemotherapy-cohort according to subgroups of TILs and PD-L1 status

*SP142*	**IDFS** (events = 42) HR (95% CI)	p- value	**OS** (events = 33) HR (95% CI)	p- value	**DRFI** (events = 28) HR (95% CI)	p- value
*TIL-high/PD-L1-pos*	*Ref*		*Ref*		*Ref*	
TIL-high/PD-L1-neg	1.75 (0.45–6.77)	0.417	1.17 (0.24–5.64)	0.845	2.46 (0.59–10.32)	0.217
TIL-low/PD-L1-pos	3.43 (0.93–6.39)	0.072	1.67 (0.58–4.76)	0.339	2.06 (0.63–6.76)	0.232
TIL-low/PD-L1-neg	3.45 (1.47–8.07)	0.004	2.61 (1.08–6.30)	0.033	3.00 (1.08–8.33)	0.035
-						
TIL-high/PD-L1-pos	0.29 (0.12–0.68)	0.004	0.38 (0.16–0.92)	0.033	0.33 (0.12–0.93)	0.035
TIL-high/PD-L1-neg	0.51 (0.15–1.70)	0.271	0.45 (0.10–1.94)	0.283	0.82 (0.24–2.86)	0.758
TIL-low/PD-L1-pos	0.71 (0.33–1.49)	0.360	0.64 (0.26–1.54)	0.319	0.67 (0.26–1.79)	0.443
*TIL-low/PD-L1-neg*	*Ref*		*Ref*		*Ref*	
-						
**22C3**	-					
*TIL-high/PD-L1-pos*	*Ref*		*Ref*		*Ref*	
TIL-high/PD-L1-neg	1.03 (0.13–8.09)	0.981	1.22 (0.15–9.72)	0.854	1.31 (0.16–10.63)	0.802
TIL-low/PD-L1-pos	2.23 (0.91–5.49)	0.081	1.79 (0.65–4.94)	0.260	1.43 (0.46–4.51)	0.539
TIL-low/PD-L1-neg	2.83 (1.30–6.14)	0.009	2.37 (1.03–5.52)	0.043	2.44 (0.99–5.98)	0.052
-						
TIL-high/PD-L1-pos	0.35 (0.16–0.77)	0.009	0.42 (0.18–0.98)	0.043	0.41 (0.17–1.01)	0.052
TIL-high/PD-L1-neg	0.36 (0.05–2.69)	0.321	0.51 (0.07–3.84)	0.514	0.54 (0.07–4.06)	0.546
TIL-low/PD-L1-pos	0.79 (0.37–1.67)	0.363	0.75 (0.31–1.82)	0.529	0.59 (0.21–1.62)	0.303
*TIL-low/PD-L1-neg*	*Ref*		*Ref*		*Ref*	

*Ref.:* Either the TIL-high/PD-L1-pos groups (which tend to have the best prognosis) or the TIL-low/PD-L1-neg groups (which tend to have the worst prognosis) are set as references. TILs: High < 30% and Low ≥ 30% stromal TIL abundance. Positive PD-L1: SP142 IC ≥ 1%, 22C3 CPS ≥ 1. Negative PD-L1: SP142 IC < 1%, 22C3 CPS < 1. Corresponding Kaplan–Meier curves are shown in Fig. 4

### Hierarchical clustering of different IHC PD-L1 categories

**Fig. 5:**
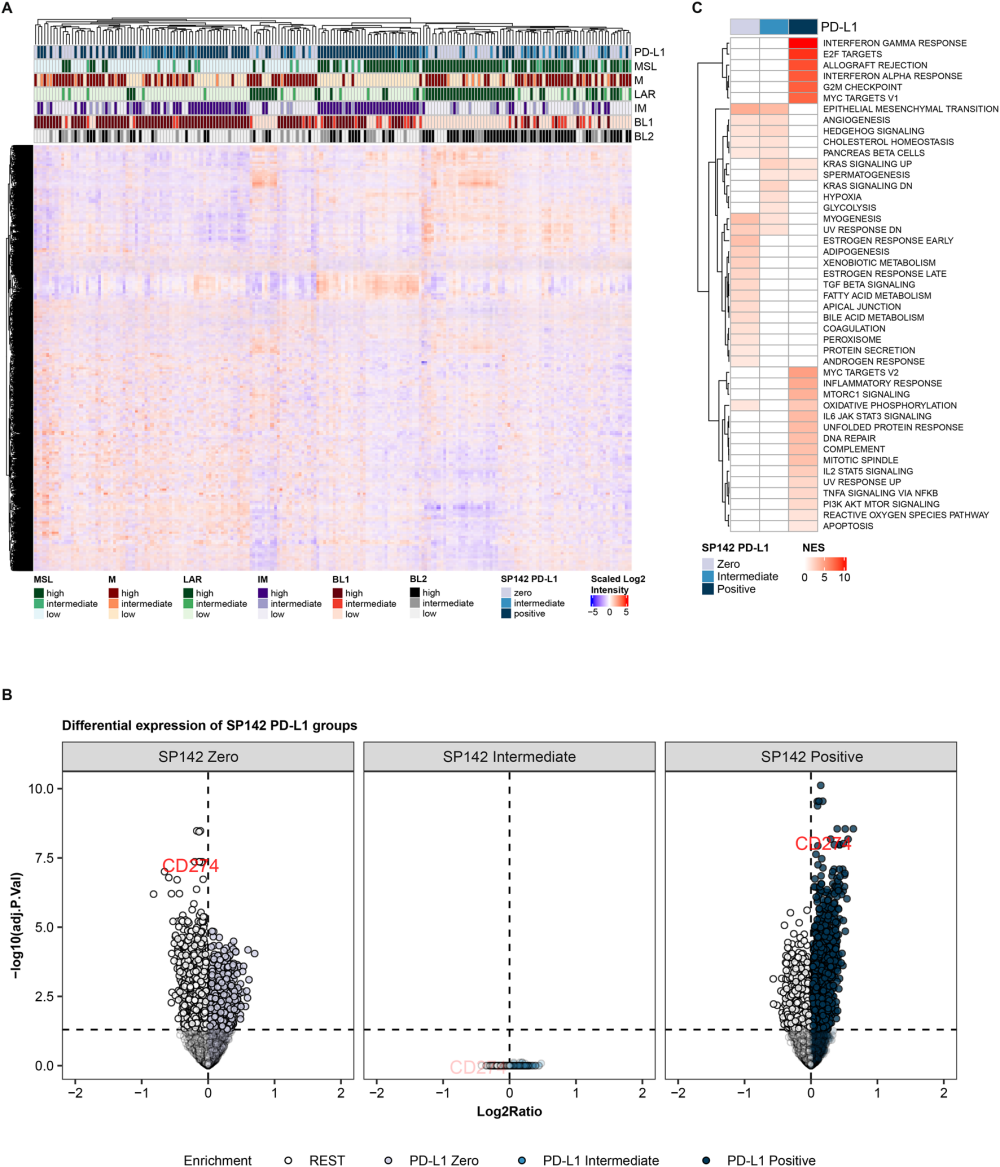
Hierarchical cluster analysis, differential expression (DE), and Gene Set Enrichment Analysis (GSEA) according to the three SP142 immunohistochemical (IHC) PD-L1 categories in the overall gene expression cohort (*N* = 194). Cluster analysis shown as a heatmap of hierarchical clustering in (**A**) and corresponding tables of cluster categorization are shown in Table 4. In (**B**) DE of genes displayed as volcano plots where each IHC PD-L1 group is compared to the other two (i.e., the “rest”). In (**C**) GSEA showing significantly enriched hallmark molecular pathways across PD-L1 categories. Each cell in the GSEA heatmap represents a pathway and if colored in red (normalized enrichment scores (NES)) it is significantly enriched compared to the other two PD-L1 subgroups, with various levels of enrichment (darker shade of red). Abbreviations: BL1 and *BL2* Basal-like 1 and 2, *IM* Immunomodulatory, *LAR* Luminal androgen receptor, *M* Mesenchymal, *MSL* Mesenchymal stem-like

In the overall cohort, a unique clustering across the three IHC PD-L1 categories was observed. In support of this, the clustering was similar for both SP142 and 22C3 antibodies. Table 4 shows exact frequencies of clustering and p values and Fig. 5A and Fig. S1A the corresponding hierarchical clustering of samples (heatmap) for SP142 and 22C3, respectively.

**Table 4:** Associations between PD-L1 IHC categories and TNBC GEX subtypes (by hierarchical clustering)

	**SP142** PD-L1 (row %)				**22C3** PD-L1 (row %)			
	IM-Low	IM- Intermed	IM-High	*p* value	IM-Low	IM-Intermed	IM-High	*p* value
Zero	49 (79.0)	7 (11.3)	6 (9.7)		38 (88.4)	4 (9.3)	1 (2.3)	
Intermediate	15 (53.6)	6 (21.4)	7 (25.0)		23 (51.1)	9 (20.0)	13 (28.9)	
Positive	21 (20.2)	15 (14.4)	68 (65.4)		24 (22.6)	15 (14.2)	67 (63.2)	
				< 0.001				< 0.001
	M-Low	M-Intermed	M-High		M-Low	M-Intermed	M-High	
Zero	12 (19.4)	6 (9.7)	44 (71.0)		5 (11.6)	2 (4.7)	36 (83.7)	
Intermediate	10 (35.7)	3 (10.7)	15 (53.6)		14 (31.1)	10 (22.2)	21 (46.7)	
Positive	57 (54.8)	12 (11.5)	35 (33.7)		60 (56.6)	9 (8.5)	37 (34.9)	
				< 0.001				< 0.001
	LAR-Low	LAR-Intermed	LAR-High		LAR-Low	LAR-Intermed	LAR-High	
Zero	23 (37.1)	7 (11.3)	32 (51.6)		13 (30.2)	6 (14.0)	24 (55.8)	
Intermediate	17 (60.7)	4 (14.3)	7 (25.0)		25 (55.6)	6 (13.3)	14 (31.1)	
Positive	78 (75.0)	7 (6.7)	19 (18.3)		80 (75.5)	6 (5.7)	20 (18.9)	
				< 0.001				< 0.001
	BL1-Low	BL1-Intermed	BL1-High		BL1-Low	BL1-Intermed	BL1-High	
Zero	38 (61.3)	7 (11.3)	17 (27.4)		29 (67.4)	8 (18.6)	6 (14.0)	
Intermediate	10 (35.7)	6 (21.4)	12 (42.9)		19 (42.2)	1 (2.2)	25 (55.6)	
Positive	22 (21.2)	16 (15.4)	66 (63.5)		22 (20.8)	20 (18.9)	64 (60.4)	
				< 0.001				< 0.001
	MSL-Low	MSL-Intermed	MSL-High		MSL-Low	MSL-Intermed	MSL-High	
Zero	19 (30.6)	5 (8.1)	38 (61.3)		11 (25.6)	5 (11.6)	27 (62.8)	
Intermediate	14 (50.0)	3 (10.7)	11 (39.3)		21 (46.7)	4 (8.9)	20 (44.4)	
Positive	54 (51.9)	12 (11.5)	38 (36.5)		55 (51.9)	11 (10.4)	40 (37.7)	
				0.039				0.056
	BL2-Low	BL2-Intermed	BL2-High		BL2-Low	BL2-Intermed	BL2-High	
Zero	19 (30.6)	15 (24.2)	28 (45.2)		14 (32.6)	11 (25.6)	18 (41.9)	
Intermediate	12 (42.9)	4 (14.3)	12 (42.9)		15 (33.3)	12 (26.7)	18 (40.0)	
Pos	42 (40.4)	25 (24.0)	37 (35.6)		44 (41.5)	21 (19.8)	41 (38.7)	
				0.522				0.758

Overall GEX cohort, *N* = 194. The corresponding heatmaps of hierarchical clustering are shown in Fig. 5A for the SP142 IHC assay and in Fig. S1A for 22C3. Abbreviations: GEX: gene expression; IHC: immunohistochemical; TNBC: triple-negative breast cancer; BL1 and BL2: Basal-like 1 and 2, IM: Immunomodulatory, LAR: Luminal androgen receptor, M: Mesenchymal, MSL: Mesenchymal stem-like

We found that PD-L1 positive tumors clustered significantly more often as Immunomodulatory (IM)-high. 65.4% and 63.2% of tumors in the SP142- and 22C3-positive groups were IM-high, respectively, compared to 9.7% and 2.3% in the SP142- and 22C3-zero groups, respectively (p value < 0.001 for both SP142 and 22C3). PD-L1 positive tumors also clustered significantly more often as Basal-Like 1 (BL1)-high (p values < 0.001).

PD-L1-zero tumors clustered significantly more often as Luminal androgen receptor (LAR)-high (p values < 0.001), Mesenchymal (M)-high (p values < 0.001), and Mesenchymal Stem Like (MSL)-high (p values 0.039 and 0.056 for SP142 and 22C3, respectively).

The PD-L1-intermediate tumors did not conclusively cluster as zero or positive tumors, although tending to cluster either as BL1-high, like the 22C3-positive tumors, or as MSL-low, like the SP142- and 22C3-positive tumors. The Basal-Like 2 (BL2) subtype was not significantly associated with PD-L1 categories.

### Differential expression and GSEA according to IHC PD-L1 categories

Differential expression of genes across the three SP142 and 22C3 PD-L1 IHC categories in the overall cohort revealed that the PD-L1-zero and PD-L1-positive subgroups differed the most in gene expression. The intermediate PD-L1 group showed similarity with both the PD-L1-positive and the PD-L1-zero group, with few or any significant gene expression differences for either group (Fig. 5B and Fig. S1B).

Gene Set Enrichment Analysis (GSEA) revealed that the PD-L1 positive tumors had 23 significantly enriched hallmark pathways compared to the other PD-L1 groups. These specifically included enrichment of immune- or inflammatory-related signaling pathways (e.g., interferon (IFN) γ and α response, allograft rejection, inflammatory response, and IL6-JAK-STAT3 signaling pathways) and gene sets involved in cell cycle and proliferation (e.g., E2F target, G2M checkpoint, MYC v1 and v2 targets and mitotic spindle signaling pathways; Fig. 5C and Fig. S1C). PD-L1-zero tumors had 20 and 15 significantly enriched pathways for SP142 and 22C3, respectively, e.g., pathways involved in cell growth, differentation, and metastatic potential (e.g., epithelial mesenchymal transition (EMT), TGF-β, estrogen responses, angiogenesis and hedgehog signaling). The PD-L1-intermediate groups categorized more like the PD-L1-zero groups than the PD-L1-positive groups based on GSEA reported enrichments; the intermediate PD-L1 tumors had 12 and 22 significantly enriched pathways for SP142 and 22C3, respectively, where 7 and 13 were also enriched in the PD-L1-zero tumors (e.g., EMT and angiogenesis), and 3, respective 6, not significantly enriched in the other SP142 and 22C3 categories (Fig. 5C and Fig. S1C).

## Discussion

Clinical effect of atezolizumab and pembrolizumab in the early-stage TNBC setting has been found in phase III trials across PD-L1 status at SP142 IC 1% and 22C3 CPS 1 thresholds, respectively [7–9]. To avoid treating the wrong patients with checkpoint inhibitors, a treatment with potential harmful side-effects, and to make it more efficient, it is of clinical interest to search for TNBC responders, and non-responders, in better defined SP142 and 22C3 categories. We therefore here evaluated alternative PD-L1 levels in a population-based cohort of 237 early-stage TNBC tumors by PD-L1 IHC staining using SP142 IC and 22C3 CPS. We defined an intermediate IHC PD-L1 group (SP142 IC > 0%, < 1%; 22C3 CPS > 0, < 1) that also was supported by intermediate PD-L1 (*CD274*) gene expression levels. The intermediate IHC groups defined by both antibody assays tended to have better prognosis than the PD-L1-zero groups and rather showed a tendency to prognostically resemble the PD-L1-positive groups.

Our prognostic findings were evident despite lower median TIL abundance in the intermediate groups than the positive groups, where TIL abundance in the PD-L1 intermediate subgroups were closer to that of the PD-L1-zero groups. This is particularly interesting given that TILs are known to be a strong positive prognostic marker in TNBC [17, 21] and might suggest that the prognostic value of PD-L1 reaches beyond the association with TILs. Indeed, we found that some PD-L1 IHC expression, even though it was < 1, had a tendency for association with positive prognostic impact. It might be relevant to search for early-stage TNBC non-responders to atezolizumab and pembrolizumab in the SP142 and 22C3 IHC zero category, respectively. Searching for potential atezolizumab responders in the SP142-intermediate group might be relevant in the metastatic setting since the predictive value here is SP142 IC ≥ 1% [3, 6]. Finding potential pembrolizumab responders in the 22C3 CPS > 0 but < 1 category in the metastatic setting is not relevant since the predictive threshold here is CPS ≥ 10 [4, 5].

We have previously reported that PD-L1 expression at more traditional thresholds (SP142 IC < 1% versus ≥ 1% and 22C3 CPS < 1 vs ≥ 1) did not remain independently positively prognostic when adjusting for TILs in multivariable regression analyses [19]. This suggested that the positive prognostic impact of PD-L1 expression might depend upon the presence of TILs. In our current study based on the same cohort, we found that cases that were low in TILs and also expressed PD-L1 < 1 tended to have poorer prognosis than cases low in TILs with PD-L1 ≥ 1, despite these groups having similar TIL abundance. Again, this indicates that the prognostic impact of PD-L1 might reach beyond the association with TILs. It might be of interest in the metastatic setting to find potential responders to atezolizumab in this TIL-low/PD-L1 < 1 group with the poorest prognosis. For example, 16 of 60 patients in this group were SP142 PD-L1-intermediate and of interest to evaluate if they might be responders.

To investigate whether the TNBC tumors clustered with unique molecular signatures, we performed GEX subtyping according to Lehmann et al. [13] and found that PD-L1 positive tumors were significantly more often subtyped as IM-high and BL1-high and PD-L1-zero tumors more often as LAR-high, M-high, and MSL-high. It has previously been suggested that the transcripts in the IM subtype might be contributed from TILs rather than the tumor cells [22] and the finding of PD-L1 positivity being associated with the IM subtype might be explained by the positive association of PD-L1 and abundance of stromal TILs. Our results are in line with previous findings where PD-L1 positivity has been found to be associated with the IM subtype and the BL1 subtype found to be associated with higher rate of immune cell infiltrates. Similarly, the LAR subtype has previously been associated with lower rate of immune cell infiltrates and the M subtype has been found to be associated with absence of immune cells and low PD-L1 expression [23–25]. We also found by using GSEA that PD-L1 positivity was associated with enrichment of immune- or inflammatory-related signaling pathways and pathways involved in cell cycle and proliferation. PD-L1 negative tumors on the other hand were associated with pathways involved in cell growth, differentation, migration, and metastatic potential. There is an overlap between the TNBC molecular subtypes and GSEA where the IM subtype has been found to be characterized by expression of genes encoding cytokines and immune antigens, BL1 by expression of genes involved in the cell cycle and DNA damage response, LAR by androgen receptor signaling, and the M subtype characterized by increased expression of genes involved in EMT and growth factor pathways [13, 22]. Our results are consistent with this in the context of PD-L1 status and with previously reported correlation of PD-L1 expression in breast cancer with cytotoxic immune response genes and immune-related pathways and features (e.g., IFN-α, IFN-γ, STAT3, and TNFα) [26, 27]. The positive correlation of PD-L1 and TIL abundance, and the observed association of PD-L1 positivity with the IM cluster and immune- or inflammatory-related pathways, is likely mediated through tumoricidal TILs who have previously been associated with mediators, e.g., IFN-γ, that induce PD-L1 upregulation [28]. Indeed, we found that the IFN-γ response pathway was one of the most enriched in PD-L1 positive tumors. The observed positive prognostic value of PD-L1 may seem paradoxical in the light of its role in tumoral immune evasion and could be explained by that it may not be an isolated immunosuppressive process but reflecting an upregulation following an ongoing cytotoxic antitumor immune response [29, 30]. The observed poorer prognosis of PD-L1 negative or TIL-low TNBC tumors might be mediated through tumor-associated macrophages and immunosuppressive myeloid-derived suppressor cells, which are tumor promoting and involved in therapeutic resistance, angiogenesis, secretion of immunosuppressive mediators, such as TGF-β, and in inducing EMT [31–33]. This is consistent with our GEX findings in the PD-L1 negative tumors. An association of EMT and PD-L1 upregulation has previously been reported, which our results are not consistent with, but might be explained through the previously described complex bidirectional regulation between these factors leading in the end to tumoral immune evasion and invasion [33].

A caveat of our study is the limited number of patients and events in each IHC PD-L1 category and TIL/PD-L1 subgroups and our results should therefore be interpreted with precaution. Another limitation is the usage of TMA cores for PD-L1 evaluation which are smaller than clinical tumor samples. Moreover, our study is retrospective and non-randomized consisting of patients diagnosed from 2010 to 2015 where administered treatment did not include checkpoint inhibitors, and therefore, we are not able to investigate the predictive value of PD-L1 or TILs.

TNBC tumors with zero (SP142 IC 0%; 22C3 CPS 0) and intermediate (IC > 0%, < 1%; CPS > 0, < 1) PD-L1 IHC expression are traditionally grouped together as PD-L1-negative (IC < 1%; CPS < 1). In summary, our study showed that TNBC tumors with intermediate IHC PD-L1 expression were also intermediate on the PD-L1 gene expression level, did not consequently categorize with PD-L1-zero tumors on the hierarcial clustering level, and tended to have better prognosis than PDL1-zero patients. Together, this suggests that the IHC intermediate PD-L1 group seems to be supported on the molecular level and that minimal PD-L1 IHC expression might have positive prognostic impact. Our findings warrant further investigation of the generally accepted threshold of PD-L1 positivity in TNBC and evaluation of the treatment predictive value at the zero cut-off level in the clinical setting, with the aim of attempting to identify potential checkpoint responders, and non-responders, among cases with IHC PD-L1 staining < 1.

## Supplementary Information

Below is the link to the electronic supplementary material.

Supplementary material 1 (DOCX 19 kb)

Supplementary material 2 (DOCX 21 kb)

Supplementary material 3 (DOCX 56,932 kb)

Supplementary material 4 (DOCX 39 kb)

## Data Availability

The datasets used and/or analyzed are available from the corresponding author on reasonable request (clinicopathological features and histological PD-L1 and TIL scoring also available in Table S3).
