# Supplementary material for: Evaluation of alternative prognostic thresholds for SP142 and 22C3 immunohistochemical PD-L1 expression in triple-negative breast cancer: results from a population-based cohort
Source: Breast Cancer Res Treat. 2024 Dec 10;210(2):271–84. doi: 10.1007/s10549-024-07561-x (PMC11930886; doi:10.1007/s10549-024-07561-x)
Supplement: Supplementary file 1 — Supplementary material 1 (DOCX 19 kb) [file 10549_2024_7561_MOESM1_ESM.docx]

| **Table S1** Clinicopathological characteristics in the cohort receiving (neo)adjuvant chemotherapy (CT) and the cohort not receiving CT | | | | |
| --- | --- | --- | --- | --- |
| n (% of known) | **CT-cohort**, N=171 | **Non-CT-cohort**, N=66 | **p-value** |  |
| **Age at diagnosis** (years) |  |  |  | |
| Median (range) | 55 (26-76) | 80 (38-91) | <0.001 | |
| <50 | 53 (31.0) | 3 (4.5) | <0.001 | |
| 50-75 | 117 (68.4) | 17 (25.8) |  | |
| >75 | 1 (0.6) | 46 (69.7) |  | |
| **Tumor size** |  |  |  | |
| ≤20 mm | 83 (53.9) | 30 (45.5) | 0.251 | |
| >20 mm | 71 (46.1) | 36 (54.5) |  | |
| Unknown | 17 | 0 |  | |
| **Lymph node status** |  |  |  | |
| Node negative (N0) | 101 (59.8) | 45 (69.2) | 0.181 | |
| Node positive (N+) | 68 (40.2) | 20 (30.8) |  | |
| Unknown | 2 | 1 |  | |
| **Nottingham histologic grade** |  |  |  | |
| 1 | 0 | 0 |  | |
| 2 | 14 (8.6) | 16 (24.2) | 0.002 | |
| 3 | 148 (91.4) | 50 (75.8) |  | |
| Unknown | 9 | . |  | |
| **Ki-67 proliferation marker** |  |  |  | |
| ≤30% | 22 (12.9) | 19 (29.2) | 0.003 | |
| >30% | 148 (87.1) | 46 (70.8) |  | |
| Unknown | 1 | 1 |  | |
| **Histological type** |  |  |  | |
| Invasive ductal carcinoma | 136 (79.5) | 51 (77.3) | 0.003 | |
| Medullary features | 17 (9.9) | 0 |  | |
| Other | 18 (10.5) | 15 (22.7) |  | |
| **TIL abundance** |  |  |  | |
| Median %, (range) | 20 (0-90) | 10 (0-100) | 0.021 | |
| <30% | 96 (56.5) | 45 (69.2) | 0.074 | |
| ≥30% | 74 (43.5) | 20 (30.8) |  | |
| Unknown | 1 | 1 |  | |
| **SP142 IC+** |  |  |  | |
| 0% | 49 (28.7) | 32 (48.5) | 0.010 | |
| >0%, <1% | 26 (15.2) | 10 (15.2) |  | |
| ≥1% | 96 (56.1) | 24 (36.4) |  | |
| **22C3 CPS** |  |  |  | |
| 0 | 32 (19.3) | 22 (33.3) | 0.043 | |
| >0, <1 | 36 (21.7) | 17 (25.8) |  | |
| 1-9 | 51 (30.7) | 11 (16.7) |  | |
| ≥10 | 47 (28.3) | 16 (24.2) |  | |
| Unevaluable | 5 | 0 |  | |
| **Death** (OS event) | 33 (19.3) | 21 (31.8) | 0.039 | |
| **Relapse** (all, distant or locoreg.) | 34 (19.9) | 15 (22.7) | 0.579 | |
| **Relapse or death** (IDFS event) | 42 (24.6) | 27 (40.9) | 0.014 | |
| **Distant relapse** (DRFI event) | 28 (16.4) | 11 (16.7) | 0.924 | |
