# Supplementary material for: Evaluation of alternative prognostic thresholds for SP142 and 22C3 immunohistochemical PD-L1 expression in triple-negative breast cancer: results from a population-based cohort
Source: Breast Cancer Res Treat. 2024 Dec 10;210(2):271–84. doi: 10.1007/s10549-024-07561-x (PMC11930886; doi:10.1007/s10549-024-07561-x)
Supplement: Supplementary file 2 — Supplementary material 2 (DOCX 21 kb) [file 10549_2024_7561_MOESM2_ESM.docx]

| **Table S2** Clinicopathological characteristics in the overall cohort according to PD-L1 status | | | | | | | | |
| --- | --- | --- | --- | --- | --- | --- | --- | --- |
|  | **SP142** (n=237) | | |  | **22C3 (**n=232) | | |  |
| n (%) | **0%**  n=81 (34.2%) | **>0%, <1%**  n=36 (15.2%) | **≥1%**  n=120 (50.1%) | **p-value** | **0**  n=54 (23.3%) | **>0, <1**  n=53 (22.8%) | **≥1**  n=125 (53.9%) | **p-value** |
| **Age at diagnosis** |  |  |  |  |  |  |  |  |
| Median (range) | 66 (26-91) | 59.5 (36-85) | 56.5 (28-90) | <0.001 | 68.5 (26-91) | 61 (27-88) | 57 (28-91) | 0.003 |
| <50 y | 12 (14.8) | 11 (30.6) | 33 (27.5) | 0.008 | 7 (13.0) | 12 (22.6) | 35 (28.0) | 0.041 |
| 50-75 y | 43 (53.1) | 20 (55.6) | 71 (59.2) |  | 29 (53.7) | 31 (58.5) | 71 (56.8) |  |
| >75 y | 26 (32.1) | 5 (13.9) | 16 (13.3) |  | 18 (33.3) | 10 (18.9) | 19 (15.2) |  |
| **Tumor size** |  |  |  |  |  |  |  |  |
| ≤20 mm | 34 (42.0) | 18 (50.0) | 61 (50.8) | 0.583 | 25 (51.0) | 27 (51.9) | 61 (51.3) | 0.995 |
| >20 mm | 38 (46.9) | 13 (36.1) | 56 (46.7) |  | 24 (49.0) | 25 (48.1) | 58 (48.7) |  |
| Unknown | 9 (11.1) | 5 (13.9) | 3 (2.5) |  | 5 | 1 | 6 |  |
| **Lymph nodes** |  |  |  |  |  |  |  |  |
| N0 | 51 (63.0) | 20 (55.6) | 75 (62.5) | 0.781 | 36 (67.9) | 29 (55.8) | 80 (64.0) | 0.467 |
| N+ | 29 (35.8) | 15 (41.7) | 44 (36.7) |  | 18 (34.0) | 23 (44.2) | 45 (36.0) |  |
| Unknown | 1 (1.2) | 1 (2.8) | 1 (0.8) |  | 1 | 1 | . |  |
| **Histologic grade** |  |  |  |  |  |  |  |  |
| 2 | 19 (23.5) | 5 (13.4) | 6 (5.0) | <0.001 | 16 (31.4) | 9 (17.0) | 5 (4.1) | <0.001 |
| 3 | 57 (70.4) | 28 (77.8) | 113 (94.2) |  | 35 (68.6) | 44 (83.0) | 117 (95.9) |  |
| Unknown | 5 (6.2) | 3 (8.3) | 1 (0.8) |  | 3 | . | 3 |  |
| **Ki-67** |  |  |  |  |  |  |  |  |
| ≤30% | 24 (29.6) | 5 (13.9) | 12 (10.0) | <0.001 | 18 (34.0) | 12 (23.1) | 11 (8.8) | <0.001 |
| >30% | 55 (67.9) | 31 (86.1) | 108 (90.0) |  | 35 (66.0) | 40 (76.9) | 114 (91.2) |  |
| Unknown | 2 (2.5) | 0 | 0 |  | 1 | 1 | . |  |
| **Histological type** |  |  |  |  |  |  |  |  |
| IDC | 60 (74.1) | 31 (86.1) | 96 (80.0) | <0.001 | 39 (72.2) | 43 (81.1) | 101 (80.8) | <0.001 |
| Medullary feat. | 1 (1.2) | 1 (2.8) | 15 (12.5) |  | 0 | 1 (1.9) | 15 (12.0) |  |
| Other | 20 (24.7) | 4 (11.1) | 9 (7.5) |  | 15 (27.8) | 9 (17.0) | 9 (7.2) |  |
| **TIL abundance**, % |  |  |  |  |  |  |  |  |
| Median (range) | 10 (0-50) | 20 (1-70) | 40 (0-100) | <0.001 | 5 (0-50) | 10 (0-50) | 40 (1-100) | <0.001 |
| <30% | 73 (90.1) | 23 (63.9) | 45 (37.5) | <0.001 | 52 (98.1) | 43 (81.1) | 44 (35.5) | <0.001 |
| ≥30% | 7 (8.6) | 13 (36.1) | 74 (61.7) |  | 1 (1.9) | 10 (18.9) | 80 (64.5) |  |
| Unknown | 1 (1.2) | 0 | 1 (0.8) |  | 1 | . | 1 |  |
