# Supplementary material for: Evaluation of alternative prognostic thresholds for SP142 and 22C3 immunohistochemical PD-L1 expression in triple-negative breast cancer: results from a population-based cohort
Source: Breast Cancer Res Treat. 2024 Dec 10;210(2):271–84. doi: 10.1007/s10549-024-07561-x (PMC11930886; doi:10.1007/s10549-024-07561-x)
Supplement: Supplementary file 3 — Supplementary material 3 (DOCX 56,932 kb) [file 10549_2024_7561_MOESM3_ESM.pdf]

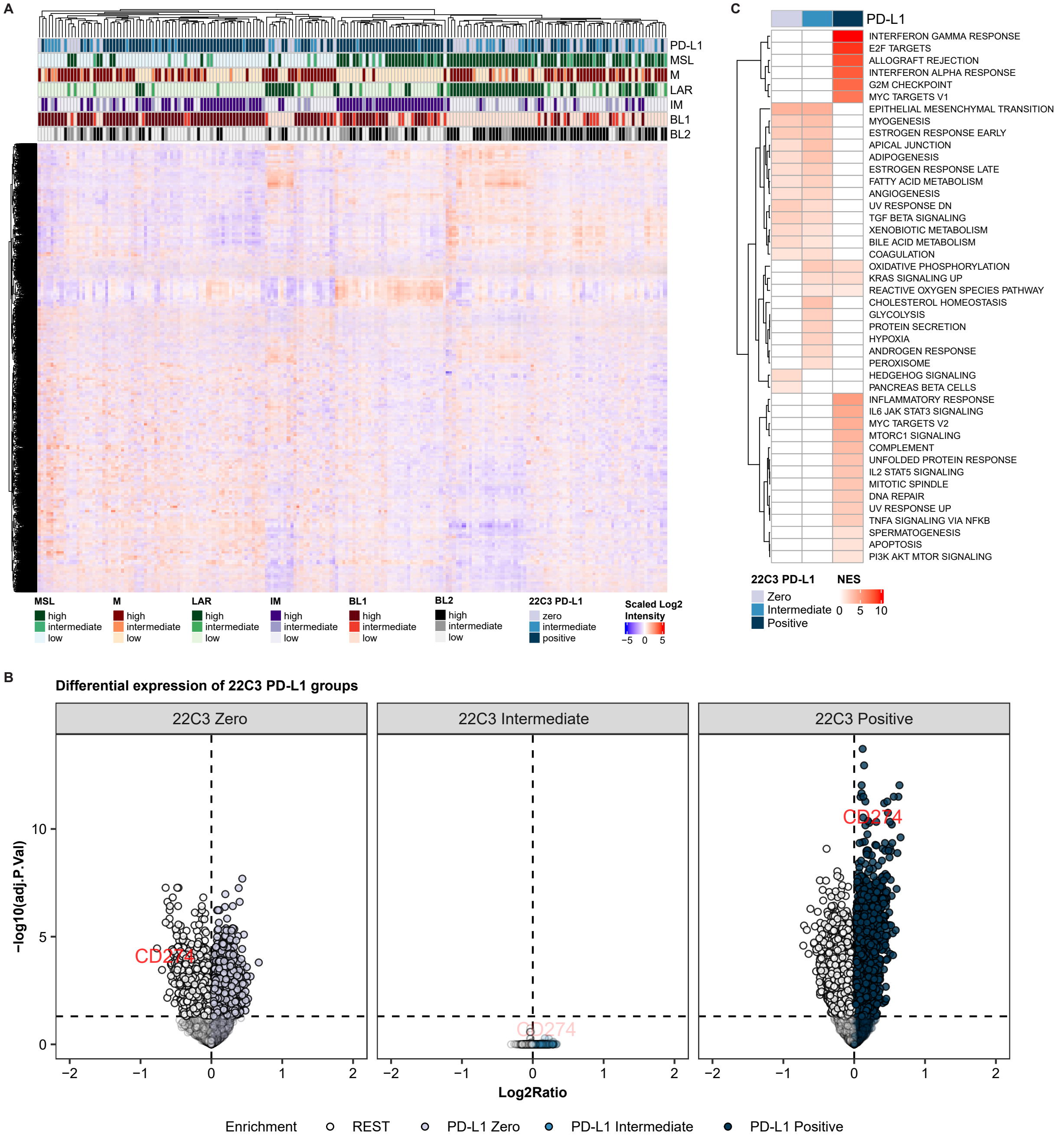

Fig. S1 Hierarchical cluster analysis, differential expression (DE) and Gene Set Enrichment Analysis (GSEA) according to the three 22C3 immunohistochemical (IHC) PD-L1 categories in the overall gene expression cohort (N=194). Cluster analysis shown as a heatmap of hierarchical clustering in **(A)** and corresponding tables of cluster categorization are shown in Table 4. In **(B)** DE of genes displayed as volcano plots where each IHC PD-L1 group is compared to the other two (i.e. the "rest"). In **(C)** GSEA showing significantly enriched hallmark molecular pathways across PD-L1 categories. Each cell in the GSEA heatmap represents a pathway and if coloured in red (normalized enrichment scores (NES)) it is significantly enriched compared to the other two PD-L1 subgroups, with various levels of enrichment (darker shade of red). Abbreviations: BL1 and BL2: Basal-like 1 and 2, IM: Immunomodulatory, LAR: Luminal androgen receptor, M: Mesenhymal, MSL: Mesenchymal stem-like.
